# Potential Spectrum of Accompanied Penetrating Abdominal Intraperitoneal Injuries with Bowel Evisceration: Surprises Awaiting the Trauma Surgeon in Resource Limited Settings

**DOI:** 10.1155/2022/8015067

**Published:** 2022-10-25

**Authors:** Ahmed Shabhay, Zarina Shabhay, Kondo Chilonga, Theresia Mwakyembe, David Msuya, Fabian Massaga, Samwel Chugulu

**Affiliations:** ^1^Department of General Surgery, Kilimanjaro Christian Medical University College (KCMUCo), P.O. Box 2240 Moshi, Tanzania; ^2^Department of General Surgery, Kilimanjaro Christian Medical Centre (KCMC), P.O. Box 3010 Moshi, Tanzania; ^3^Military Hospital Mwanza, P. O Box 589 Mwanza, Tanzania; ^4^Muhimbili Orthopedic Institute (MOI), P.O. Box 65474 Dar es Salaam, Tanzania; ^5^Bugando Medical Centre, P. O Box 1370 Mwanza, Tanzania

## Abstract

Penetrating abdominal injuries involves violation of the peritoneal cavity and injuries to solid organs and other intraperitoneal viscera such as major blood vessels and hollow organs. Typically such injuries arise from gunshot wounds or stab wounds. With increase in crime rates and motor traffic accidents in urban areas, the trauma surgeon in civilian urban centers faces spectrum of injuries similar to his colleague in war torn areas. Potential spectrum of penetrating abdominal injuries is wide and accurate diagnosis in resource limited centers is challenging. Majority of injuries are concealed and diagnosed intraoperatively and dealt with relatively junior trauma surgeons in emergency settings in remote limited settings. Computed tomography (CT) scans and Magnetic Resonance Imaging (MRI) facilities are scarce in resource limited settings. Haemodynamic states of penetrating abdominal injuries patients presenting in emergency departments necessitate urgent surgical exploration and management with minimal room for full radiological work-up. Evisceration of bowels with unstable haemodynamic states mandate laparotomy due to wide spectrum of accompanied intraperitoneal injuries. Four cases of penetrating abdominal injuries are presented with modes of assault ranging from gunshot injuries to stab wounds with broken bottles to highlight the intra-abdominal spectrum of injuries, challenges in diagnosis and emergency managements done in a resource limited setting.

## 1. Introduction

The abdomen is the most injured area in trauma patients [1], with abdominal trauma leading in morbidity and mortality in all age groups in the world [2, 3]. However, identifying life threatening intraperitoneal injuries due to trauma is a challenge [2]. Stab wounds and gunshot wounds are the most common causes of penetrating abdominal injuries [3–6]. Stab wounds are more common but with low mortality rates [6, 7]. Male predominance is seen in penetrating abdominal injuries [2, 3, 8–10]. The violation of abdominal cavity results in the small bowel (50%), large bowel (40%), liver (30%), and intra-abdominal vasculature (25%) constituting the higher spectrum percentage of intraperitoneal viscera injury [4]. Intraperitoneal spectrum of injuries is unpredictable as initial primary assault maybe further complicated by secondary injuries arising from either bone or bullet fragments [4, 5]. Concealed injuries further contribute to morbidity and mortality of victims [3, 4]. Presentation of the injuries varies depending on the type of penetrating object or ballistic kinetic energy involved [4], viscera involved, and number of wounds [5]. Immediate surgical exploration is warranted in haemodynamically unstable patients presenting with hypotension, signs of poor end organ perfusion, weak pulse, tachycardia, and peritonism with high index of potential vascular injury [5]. Standard radiological preoperative assessment involves chest and abdominal roentgenography, triple contrast computed tomography scans, retrograde urethrograms, and cystograms to rule out injuries ranging from diaphragmatic involvement to bladder injuries [5]. Surgical management of patients depends on mechanism of injury, haemodynamic state, and accompanying injuries. High kinetic energy injuries such as gunshot wounds almost always necessitate exploration due to vast spectrum of accompanying intraperitoneal injuries. Stable haemodynamic stab wounds patients may not always necessitate surgery [5, 7, 11]. Four selected cases of penetrating abdominal traumas are presented to highlight the spectrum of injuries, diagnostic challenges, and emergency surgical management.

## 2. Case Presentations

### 2.1. Case One

A 26-year-old male was referred to our center with history of penetrating abdominal injury on the left lumbar region of his abdomen. He arrived at our center 12 hours post injury. The referral clinical notes stated his blood pressure was 76/48 mmHg and no urine collected on urinary bag through urethral catheter. Amount of intravenous fluids given was not documented.

On admission his Glasgow coma scale was 15/15; he was severely pale, not jaundiced, not cyanosed, ill-looking, and in-distress. His vital signs on admission were blood pressure 94/46 mmHg, pulse rate 117 beats per minute regular with weak volume, respiratory rate 22 cycles per minute, temperature 35.9°, and oxygen saturation 94% on room air. On systemic examination of other systems were normal except for the abdomen where he had oedematous gangrenous small bowel evisceration of about 12 cm long on his left lumbar abdominal area through a 2 cm diameter stab wound. He had muscle guarding and tender abdomen on palpation. On admission, the patient did not reveal the true mode of his injury and claimed he fell on bathroom tiles. His hemoglobin level was 10.4 g/dL. Blood grouping and crossmatch were done, intravenous fluids normal saline and a unit of blood were transfused, and patient was taken for emergency explorative laparotomy.

Intraoperative findings were oedematous gangrenous small bowel evisceration of about 12 cm long on his left lumbar abdominal area 2 cm diameter stab wound, massive retroperitoneal haematoma of about 2.5 liters with active oozing of blood from retroperitoneal space, multiple mesenteric lacerations with mesenteric haematoma, jejunal perforations at 80 cm from ligament of Treitz, and a longitudinal infrarenal inferior vena cava laceration about 2 cm long. The spleen, liver, gastrium, large colon, and kidneys were normal.

**Figure 1:**
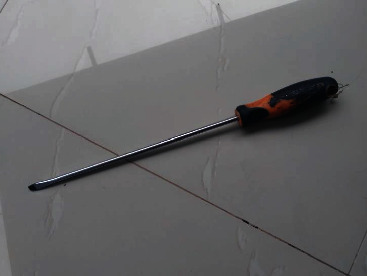
A similar standard 10-inch long screw driver used to inflict trauma in case one. As it is observed, it is long enough to transverse through anterior abdominal wall, intraperitoneal viscera up to retroperitoneal space traumatizing the IVC.

The eviscerated bowels were reduced, resected and primary anastomosis done. Lacerated jejunal segment was also resected and primary anastomosis done. Retroperitoneal haematoma was evacuated and bleeding from the inferior vena cava was controlled by applying pressure by index fingers supra and infra segment of the lacerated inferior vena cava segment and repaired by Nylon 6-0 by continuous suturing. Intraoperative blood pressure was ranging as low as 60/30 mmHg. The mesenteric lacerations were repaired by vicryl no. 2. Intraoperatively, we estimated the patient had lost about 4.5 liters of blood in total from the haematoma evacuated and bleeding from the actual surgery. There was still some diffuse oozing from the mesentery. We packed the abdomen with two abdominal mops, inserted two abdominal drains, repaired abdominal stab wound, and closed up the abdomen for continual resuscitation in intensive care unit. The surgery took 6 hrs. He received a total of 6 units of blood. Postoperatively on day one, the patient's abdominal drain totaled 900 mL of hemorrhagic fluid. No anticoagulants were instituted as prophylaxis for embolism in fear of massive intraperitoneal bleeding as not all bleeders were arrested during initial index damage control surgery. The patient improved clinically. He revealed he was stabbed by a standard 10-inch screw driver during a street quarrel (Figure 1).

On day five postoperatively, the patient had an acute onset of chest pain, developed difficulty in breathing, and succumbed to cardiac arrest. Postmortem was not performed. We suspected patient had developed pulmonary embolism which lead to his death.

### 2.2. Case Two

**Figure 2:**
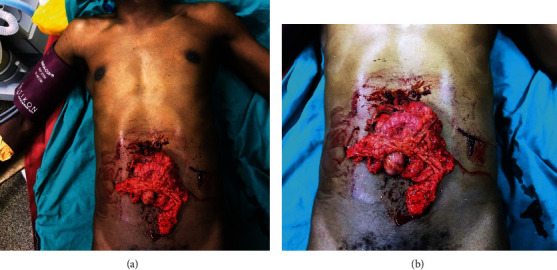
(a and b) Eviscerated omentum with transverse colon. Patient sustained penetrating abdominal injury from broken beer bottle.

41 years old man was brought at our center with history of being assaulted by a broken beer bottle in a bar fight 4 hours post injury. He was clinically stable with a blood pressure of 110/60 mmHg, pulse rate of 80 beats/min, oxygen saturation of 99%, and temperature of 36.9°C. On examination he had a Glasgow Coma Scale of 15/15, moderately pale, not jaundiced nor cyanosed. Per abdomen he had an evisceration of omentum and part of transverse colon (Figures 2(a) and 2(b)).

He was wheeled into the operating theater for emergency explorative laparotomy. Abdomen was opened through extended midline incision. Intraoperative findings were evisceration of omentum and part of the transverse colon through a 4 by 5 cm anterior abdominal wall defect, a 2 cm jejunal traumatic perforation 20 cm from ligament of Treitz, and a 1 cm transverse colon serosal tear. Bowel traumatic perforations were repaired with vicryl no. 2-0 and anterior abdominal wall defect with vicryl no 2. He received two units of whole blood. He was instituted on I/V ceftriaxone 1 g and metronidazole 500 mg 8 hrs. postoperative. Postoperative recovery was uneventful. He was discharged fourth day postoperatively. Patient was reviewed 4 weeks post discharge faring well.

### 2.3. Case Three

36 years old man was brought to our center with history of gunshot injury to the abdomen 4 hours prior admission. He was conscious, Glasgow Coma Scale 15/15, moderately pale, and oxygen saturation 99% on room air. His blood pressure was 148/67 mmHg and pulse rate of 87 beats/min. Per abdomen he had two wounds on left lumbar area 3 cm in diameter with smooth margins entry wound with blood oozing and small bowel evisceration of about 4 cm and right lumbar area, and a rouged edged wound 2 by 3 cm exit wound. Abdomen was opened through extended midline incision. Intraoperative findings were complete jejunal transections at 90 cm and 110 cm from ligament of Treitz and 0.5 cm perforation at 120 cm. The rectus abdominis muscle on left lumbar area was transected with ruptured peritoneal fascia. About 20 cm of jejunum was resected and end to end anastomosis done. Rectus abdominis muscle was repaired with its underlying fascia. Abdomen closed and he was instituted on I/V ceftriaxone 1 g and metronidazole 500 mg 8 hrs. postoperative. Postoperative recovery was uneventful.

### 2.4. Case Four

**Figure 3:**
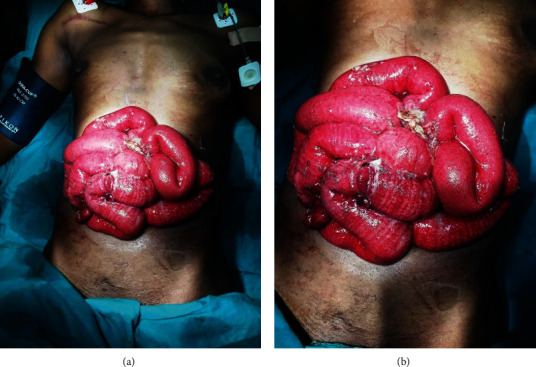
(a and b) Eviscerated small bowels with traumatic perforation in case three patient from knife stab wound.

33 years old female was admitted at our center with history of being stabbed by a knife on her abdomen. She arrived with Glasgow Coma Scale of 15/15, blood pressure 140/70 mmHg, pulse rate of 80 beats/min. Per abdomen examination, she had evisceration of small bowel from her left hypochondriac area lying on anterior abdominal wall (Figures 3(a) and 3(b)). Intraoperative findings were 3 by 3 cm defect on the left hypochondriac area, with evisceration of small bowel. Two traumatic perforations along the greater curvature approximately 2 by 2 cm on the gastrium, multiple traumatic perforations on the jejunum from 50 cm to 120 cm from the ligament of Treitz with complete transection at 100 cm mark with haemoperitoneum of approximately 300 mL. Gastric perforations were repaired with vicryl 2-0 and jejunum resected 45 cm from ligament of Treitz to 130 cm mark. Abdomen closed in layers. She was instituted on I/V ceftriaxone 1 g and metronidazole 500 mg 8 hrs. postoperative. Postoperative recovery was uneventful.

## 3. Discussions

Penetrating abdominal trauma involves external objects/ballistics violating anterior abdominal wall into the peritoneal cavity [4]. The increase in violence among the civilian populations has increased the spectrum of trauma injuries resembling those seen in war zones [5], thus making the urban trauma surgeon working in civilian populations facing similar trauma cases as his colleague in war zones. This has been due to the availability of weapons, and presence of military conflicts [4, 5]. However spectrum of injuries in war zones are different as most assaulting objects are high velocity ballistics [6] The trauma surgeon is facing a wider spectrum of intra-abdominal injuries due to a wide range of instruments/weapons available among the civilian population. Nyongole et al. found 69% of penetrating abdominal trauma patients were due to assaults [2]. The most common causes of penetrating abdominal injuries faced by the trauma surgeon are stab wounds (31%), gunshot wounds (64%), shotgun wounds (5%) [4, 5], and industrial accidents [4]. Stab wounds are encountered three more times than gunshot injuries [7] which have wider spectrum of intraperitoneal organ injuries and most often require exploration [5]. The four cases presented ranged from gunshot injury, stab wounds by knife, screw driver, and broken bottle. All cases were results from assaults and conflicts.

The pattern of intraperitoneal organs injury involves small bowel (50%), colon (40%), liver (30%), and intraperitoneal vasculature (25%) injuries [4, 5]. Close contact trauma inflicted wounds have greater kinetic energy thus inflict wider spectrum of intraperitoneal injury [4, 5]. Magnitude of injury depends on viscera affected, nature of assaulting object and the quantity of energy transmitted [4]. Spectrum of injuries can be increased by injuries from patients own bone fragments or bullets fragments [4, 5].

The pattern of injuries from the cases presented ranged from inferior vena cava injury, small bowel, gastric, and large bowel traumatic perforations. The spectrum of injuries seen, vasculature trauma was diagnosed intraoperative as seen in case one as the preoperative diagnostic radiological modalities such as Focused Assessment with Sonography in Trauma (fast) are limited in diagnosis of vessel injury. Advanced imaging technology increases diagnostic accuracy of injuries [5].

Inferior vena cava injuries are rare vascular injuries due to its retroperitoneal location and buffer from intraperitoneal organs. In our case the assaulting weapon was a long 10-inch screw driver which could transverse through the anterior abdominal wall and cushioning intraperitoneal organs to reach the inferior vena cava. It is however the most injured intra-abdominal vascular structure constituting to 25% of abdominal vascular injuries [12]. The most often injured segment of the inferior vena cava is the infrarenal segment (39%) [13]. Mortality rates are high (65%) due to insufficient fluid replacement, challenges in diagnosis, and skillset in management [13], with little improvements over the last four decades [12].

Inferior vena cava injuries are generally diagnosed intraoperatively and are accompanied with other peritoneal organs injuries with retroperitoneal haematoma [14]. This was also the observation in our case as the IVC lesion was concealed by a huge retroperitoneal haematoma. They are normally overlooked when accompanied with other intraperitoneal injuries. The first case presented, initial anticipated impression was of bowel injury due to the bowel evisceration as the patient did not reveal the true nature of the assault and assaulting weapon. IVC injury was diagnosed intraoperatively and senior surgeon consult had to be done upon discovering the spectrum of injury involving major vessels. The emergency surgery was being conducted by relatively junior surgical residents prior the intraoperative consult. The IVC laceration bleeding was discovered during peritoneal cleaning with normal saline post repair of bowel and mesenteric laceration which dislodged the haematoma concealing the injury. Damage control surgery was then done involving repairing of the IVC laceration and peritoneal cavity packing with abdominal packs. Abdomen was closed and planned for re-exploration post ICU continual resuscitation.

Diagnosing full extent of intraperitoneal injuries from clinical assessment is difficult as injuries are concealed as peritonism and hypovolemic shock may still occur in conscious individuals [4]. Standard preoperative radiological assessment includes X-rays, CT scans, and MRI due to the wide spectrum of injuries [4, 5]. However, even with clinically stable patients without any signs of peritonism might still require advance radiological investigations to diagnose concealed intraperitoneal viscera injury [4]. Preferred radiological diagnostic tool is the triple contrast CT scan [4]. This is not always available in all limited resource centers.

The standard management protocol for penetrating abdominal trauma is laparotomy [5, 15]. However, management of trauma patients poses a dilemma for attending trauma surgeons weighing the risk of missing life threating injuries when non/minimal invasive diagnostic and therapeutic procedures are opted versus the standard traditional open laparotomy method [16]. From a clinical standpoint, surgical indications for exploration are haemodynamic unstable patient, development of signs of peritonism, and unresolving diffuse abdominal pain [4]. However for patients who are haemodynamically stable, with no signs of peritonism and have been evaluated with triple contrast CT scan, laparotomy maybe withheld and observed [5, 11]. This concept was first introduced by Butt et al. as “selective conservatism” [7].

However for those cases were selective nonoperative management is opted, we opine that this should be done in controlled settings were continual reassessment and close monitoring are possible and experienced trauma surgeons are available in emergency settings if the patient's condition is to deteriorate [7]. Laparotomy is the preferred choice by surgeons working in limited resource settings [8] as there is increased potential of complications due to the wide spectrum of accompanied intraperitoneal organ injuries. Experience of the trauma surgeon in assessment of the patient as a whole entity and making clinical decisions basing on conclusions made on haemodynamic stability of the patients plays a key role in correctly selecting patients for conservative/nonoperative approach [7]. Ideally, these patients should be re-evaluated by the same trauma surgeon within the first 24-hour period [7]. Most trauma centers in limited resource settings are manned by relatively junior doctors, minimal staffing, and diagnostic tools [17]. Patients with isolated omental evisceration with benign abdomen, eviscerated omentum can safely be amputated, ligated, stump irrigated, reduced back to peritoneal cavity, and abdominal stab wound closed and observed [18, 19]. However with bowels evisceration, laparotomy is warranted [19].

There is controversy on the roles of diagnostic laparoscopy in management of penetrating abdominal injuries [20] with no consensus on its role [15] The high rate of missed injuries and development of tension-pneumothorax pose a serious risk in trauma patients [16]. Patients with haemodynamic instability, intracranial trauma, bowel evisceration, and overt peritonitis are contraindicated for laparoscopy [16]. In haemodynamic stable patients, laparoscopy can be employed for diagnosis of potential peritoneal injury when in doubt “unclear abdomen” and treatment preventing unnecessary laparotomies leading to shorter hospital stay [16]. Laparoscopic settings require trained staff and equipments [16]. Decision on laparoscopy is dependent on surgeons' experience and availability of equipment [15].

**Figure 4:**
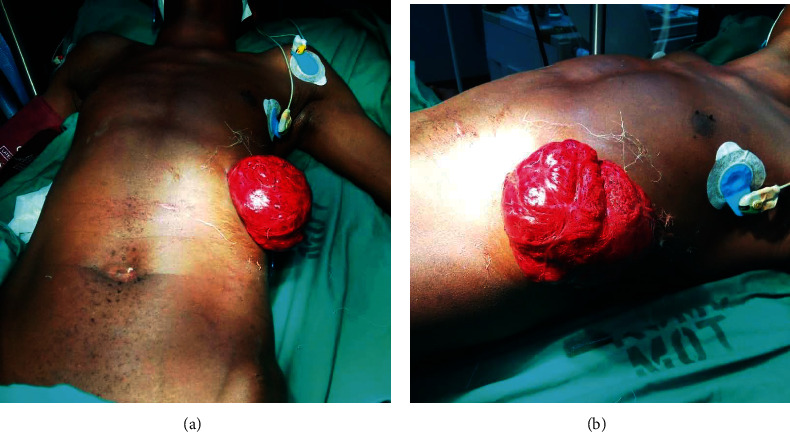
(a and b) Eviscerated omentum with bowels in a patient who sustained penetrating abdominal injury from knife stab wound on his left hypochondriac area. He also sustained diaphragmatic laceration which was repaired through abdominal approach.

Care should be taken as medication may affect blood pressure values with patients with higher pain threshold withstanding clinical signs and symptoms of peritonitis [7]. However, there is a high incidence of intraperitoneal viscera injury with patients presenting with bowels eviscerations thus mandating exploration [8, 9, 19, 21, 22]. This was the observation in all the four cases presented as all had intraperitoneal organs eviscerations and had accompanying viscera injuries which needed therapeutic surgical repair (Figures 1–4).

Bowel eviscerations occur post recent laparotomy in wound dehiscence and penetrating abdominal trauma due to slash or stab wounds to the anterior abdominal wall [23].

Kong et al. found the most commonly eviscerated organs were small bowel (70%), large bowel (26%), and gastrium 3% [8]. In our cases, three patients had small bowel evisceration with other intraperitoneal viscera injury. Only (1%) of the patients by Kong et al. had combined evisceration of more than one intraperitoneal viscera [8]. One case in our cases series had combined omentum and transverse colon evisceration. He had sustained anterior abdominal wall umbilical area broken beer bottle stab wound. However combined omental and viscera evisceration post anterior abdominal stab wound is uncommon [9]. Prognosis of intraperitoneal injuries depends on extent of trauma, time of presentation, intestinal contents peritoneal cavity contamination, haemorrhage, wide spectrum of organ injury, female sex [5], accompanying brain trauma, or bleeding disorders increases the mortality rate [4].

## 4. Conclusion

Laparotomy is the standard modality for the management of eviscerated intraperitoneal viscera in penetrating abdominal injuries. There is always accompanied injury of peritoneal viscera or the eviscerated bowels. In our cases, series reported all had presented with bowel evisceration from penetrating abdominal injury either from stab wounds or gunshot. Intraoperative findings revealed injury to the eviscerated organs with accompanied intra-abdominal viscera. Preoperative diagnosis of entire spectrum of all intraperitoneal injuries is difficult in resource limited settings as sophisticated radiological investigations are not available such as MRI and triple contrast CT scan. In our cases due to the nature of presentation of the patients having bowels eviscerations with perforations, our patients were wheeled straight into operating room for surgical interventions. Full radiological work up is not always possible in emergency limited resource set-up. In our cases, no abdominal radiological work up was ordered due to obvious nature of eviscerated bowels on anterior abdominal wall. Advocating for CT scanning/MRI imaging of abdomen would be time consuming, financially restrictive, and worsening haemodynamically state due to continual intraperitoneal hemorrhage in an otherwise obvious absolute indication for exploration. Selective nonoperative management for penetrating abdominal trauma in resource limited settings poses a challenge and risk for further complications in austere uncontrolled settings. In resource limited settings, we advocate for explorative laparotomy in eviscerated intraperitoneal viscera for surgical reduction of contents into peritoneal cavity through an extended midline incision, exploration of injury to peritoneal viscera and repair, cleaning/irrigation of violated contaminated peritoneal cavity, and repairing anterior abdominal wall defect.

## Data Availability

All data and materials pertaining to this case series can be made available on request.
